# TEN-YEAR ANALYSIS OF ADVERSE DRUG REACTIONS IN PEOPLE WITH DEMENTIA USING FDA ADVERSE EVENT REPORTING SYSTEM DATA

**DOI:** 10.1093/geroni/igae098.4291

**Published:** 2024-12-31

**Authors:** Jieyu Mao, Miyae Yamakawa, Yasushi Takeya

**Affiliations:** Osaka University, Suita, Osaka, Japan; Osaka University, Suita, Osaka, Japan; Gerontological and Geriatric Nursing Research Unit, Osaka, Osaka, Japan

## Abstract

Dementia often leads to polypharmacy and increased ADR risk, making ADR recognition vital since People with Dementia (PwD) struggle to communicate discomfort. This study used the FDA Adverse Event Reporting System (FAERS) database from 2013 to 2024 to extract dementia-related ADR reports. A total of 23,519 ADR reports were analyzed after data cleansing. Missing data was addressed using the MissForest algorithm and refined with KNN imputation. Signal detection calculated PRR (Proportional Reporting Ratio) for frequently reported drugs and ADRs. Over the ten years, the number of dementia-related ADR reports fluctuated significantly, while showing a sharp peak around 2023. Consumers are the most common reporters of ADRs, accounting for 33.4% of the reports, followed by medical doctors at 30.1%. The median time from treatment initiation to the occurrence of ADR was 20 days. Regarding patient outcomes, 38.2% reported hospitalization, and 12.4% reported death. The most reported ADRs were death (706 cases), drug ineffective (527 cases), off label use (502 cases) and aggression (458 cases). Approximately 48.8% of the top 100 ADR reports can be observed and reported by nurses. Exelon Patch was the drug most frequently reported to cause ADRs, strongly associated with death, while Epidiolex showed significant signals for seizure. Cholinesterase inhibitors and antipsychotics/anticonvulsants had high ADR report counts across multiple system organ classes, especially nervous system. Due to cognitive impairments, PwD often face challenges in reporting symptoms, highlighting the importance of objective observation and monitoring by healthcare professionals to ensure accurate ADR identification and improve PwD outcomes.

